# Groove in drum patterns as a function of both rhythmic properties and listeners’ attitudes

**DOI:** 10.1371/journal.pone.0199604

**Published:** 2018-06-29

**Authors:** Olivier Senn, Lorenz Kilchenmann, Toni Bechtold, Florian Hoesl

**Affiliations:** School of Music, Lucerne University of Applied Sciences and Arts, Lucerne, Switzerland; University of Western Ontario, CANADA

## Abstract

Music psychology defines groove as humans’ pleasureable urge to move their body in synchrony with music. Past research has found that rhythmic syncopation, event density, beat salience, and rhythmic variability are positively associated with groove. This exploratory study investigates the groove effect of 248 reconstructed drum patterns from different popular music styles (pop, rock, funk, heavy metal, rock’n’roll, hip hop, soul, R&B). It aims at identifying factors that might be relevant for groove and worth investigating in a controlled setting in the future. Drum patterns of eight bars duration, chosen from 248 popular music tracks, have been transcribed and audio reconstructions have been created on the basis of sound samples. During an online listening experiment, 665 participants rated the reconstructions a total of 8,329 times using a groove questionnaire. Results show that, among 15 tested variables, syncopation (*R*^2^ = 0.010) and event density (*R*^2^ = 0.011) were positively associated with the groove ratings. These effects were stronger in participants who were music professionals, compared to amateur musicians or mere listeners. A categorisation of the stimuli according to structural aspects was also associated with groove (*R*^2^ = 0.018). Beat salience, residual microtiming and rhythmic variability showed no effect on the groove ratings. Participants’ familiarity with a drum pattern had a positive influence on the groove ratings (*η*^2^ = 0.051). The largest isolated effect was measured for participants’ style bias (*R*^2^ = 0.123): groove ratings tended to be high if participants had the impression that the drum pattern belonged to a style they liked. Combined, the effects of style bias and familiarity (*R*^2^ = 0.152) exceeded the other effects as predictors for groove by a wide margin. We conclude that listeners’ taste, musical biographies and expertise have a strong effect on their groove experience. This motivates groove research not to focus on the music alone, but to take the listeners into account as well.

## Introduction

In music psychology, *groove* is defined as humans’ pleasurable urge to move their bodies rhythmically in response to music [1–5]. The field of groove research investigates the factors that influence this kind of musical experience.

The groove concept originally emerged within the communities of Western popular music styles like funk, soul, R&B, rock and jazz [6–8]. In this context, groove is an everyday term that can adopt a wide variety of meanings: musicians might refer to the act of playing music together as *to groove* [9]. *A groove* may mean certain aspects of the composition, a “multi-layered pattern” [10] that constitutes the basis of a tune or song (sometimes also called a *riff* or a *vamp*). Such patterns have been analysed in musicology and ethnomusicology [7, 9–16]. Finally, if musicians or listeners say that music *has groove*, they mean that the music is aesthetically pleasing to them, it does invite body movement, and creates a state of flow and effortlessness [17].

Music psychology narrowed the scope of the groove concept by defining it exclusively as the pleasurable urge to body movement triggered by music. In this very precise sense, the groove phenomenon is not limited to Western popular music, since synchronised body movement behaviour as response to music is widespread across many cultures. Whenever jazz has “swing” [9], American-Polish polka is played with “push” [18], Brazilian Choro has “balanço” [19] or Swiss folk music is played “lüpfig” [20], and listeners have the impulsion to tap their feet or bob their heads in synchrony with music, the groove phenomenon as defined by music psychology is at work [21].

In this sense, the groove experience contributes to arousal and mood regulation by definition, which have been identified as primary reasons why many humans engage with music [22]: music with groove activates our bodies and it raises our spirits, be it in dance [23–25], at the workplace [26–29], in sports [30–36], or ritual [37], regardless of musical style or cultural context [38].

*Groove* and its near relative *swing* have been a topic in musicology and ethnomusicology at least since the 1950s [39, 40]. The empirical study of groove as a research field within music psychology, however, only dates back to the early 2000s [1]. This branch of research has predominantly focused on identifying music- or stimuli-related factors that influence the groove experience in the music psychological sense, i.e. humans’ pleasurable urge to move with the rhythm of music:

- *Microtiming*: Many jazz musicians have stated [9, 17, 41] that microtiming, small temporal displacements from perfect synchrony or isochrony arising in competent performance (for a definition of microtiming, see [42, 43]), are an important factor for groove. This idea was promoted in a scholarly context by Keil [18, 40, 44, 45]. A series of studies in ethnomusicology, jazz, and popular music research showed that context-specific microtiming patterns were ubiquitous in performed jazz [46–49], Cuban music [50], Brazilian music [51–53], Norwegian folk music [54], Malian drumming [55–57], R&B, hip hop, electronic dance music [58] and in drumset playing [59–61].
  Several empirical studies investigated the effect of microtiming on the groove experience. Some reported that microtiming seemed to be of little importance for groove [3, 62, 63]. Others found that introducing microtiming deviations into otherwise metronomically precise music affected the groove experience of listeners negatively [64–66]. Yet others claimed that expert performance microtiming did not diminish entrainment or pleasure, but exaggerating the microtiming pattern had a negative effect on groove [67, 68]. One recent study [69] found that listeners preferred stimuli with small expert performance microtiming deviations to stimuli with quantised timing. In summary, there are conflicting findings concerning the influence of microtiming on groove.
- *Syncopation* arises when accented notes are played on weak positions of the underlying metrical structure [70–72]. Syncopation violates listeners’ expectations for regularity [5, 73] and it is generally seen as a form of rhythmic complexity [7] that makes rhythm more interesting to listeners. Several studies found that syncopation was a significant predictor for the groove experience: in 2014, Madison & Sioros [63] showed that musicians introduced additional syncopation into their playing if they wanted to play with high groove. Sioros et al. [73] found in two listening experiments that a medium degree of syncopation in a piano melody triggered a stronger experience of groove than no syncopation or high syncopation. This result resonates with Witek et al. [5, 74], who reported in 2014 that a medium degree of syncopation in stimuli maximised groove ratings, while low or high degrees of syncopation were associated with lower groove ratings. They suggested that the relationship between syncopation and groove followed a ∩-shaped Wundt curve, comparable to Berlyne’s [75] model describing the relationship between complexity and aesthetic appreciation in the perception of art. In a 2017 study, Witek et al. [76] monitored participants’ actual body movement response and obtained results that disagreed with their earlier results, to a certain degree: in the later study, stimuli with little or medium syncopation triggered more entrainment in listeners than stimuli with high syncopation. The study did not find further evidence for a ∩-shaped relationship between synopation and groove. To summarise, there is a general agreement that syncopation is associated with groove, but the exact nature of this relationship is unclear as of today.
- *Beat Salience*, *Event Density*, *Rhythmic Variability*, *Tempo*: Four studies explored how selected audio features were associated with groove. In 2011, Madison et al. [3] let listeners rate the groove quality of short excerpts taken from 100 commercially available audio recordings representing five different music cultures and genres. They derived acoustic and structural predictor variables from the audio signals, using signal processing methods. They found that beat salience (the acoustical markedness of the regular beat in the audio signal) and event density were positively associated with the groove ratings: music with an acoustically salient regular beat and high event density had a tendency to obtain high groove ratings. The results on beat salience were largely confirmed by Stupacher et al. in 2016 [77] who additionally found that high sound intensity in the lowest bass range was positively associated with groove. Also in 2016, Wesolowski & Hofmann [78] studied 198 excerpts from electronic dance music and found that stimuli with a non-isochronous bass and high rhythmic variability in the upper frequency domain were positively evaluated by listeners, compared to stimuli that did not show these characteristics. Finally, in a recent study, Etani et al. [79] found that the groove experience was influenced by the tempo of the music.

*Listener-related factors* and their association with groove have also been investigated in the past. But the role of these variables (as predictors for groove, as controls, or as response variables) differs across studies.

- *Expertise*: Listeners’ musical expertise was studied under the assumption that music expert listeners would be more sensitive to musical phenomena (like microtiming deviations or syncopation) than listeners with less expertise. Some studies concluded that listeners’ expertise did not influence the experience of groove within the context of their investigations [5, 62, 64, 69, 73]. Others found that musical expertise did have a moderating effect on groove: higher musical expertise was associated with a stronger effect of microtiming on the groove experience [65, 67, 68] or with physiological reactions linked to groove [80]. In their 2017 study, Witek et al. [76] observed an interaction effect between syncopation, musical expertise and musical entrainment: musicians’ body movements were better synchronised with high syncopation music than non-musicians’ movements.
- *Taste*: Musical taste or style preference is a major topic in music psychology, and, generally, taste was found to be a good predictor for a listener’s aesthetic appreciation of music (for an overview, see [81]). The role of taste has rarely been considered in the context of groove studies, and the few results were inconsistent: Butterfield [62] observed in 2010 that, in his experiment, listeners’ taste in music did not influence their perception of microtiming. Yet, Wesolowski & Hofmann [78] showed in 2016 that musical preference affected groove ratings significantly.
- *Familiarity*: The relationship between groove ratings and listeners’ familiarity with the repertoire, from which the experimental stimuli were derived, was considered in several studies. Madison et al. [3] asked participants to rate their *Familiarity* with the stimuli and found that familiarity ratings were positively correlated with the groove ratings. Janata et al. [4], in their first study, reported a tendency in listeners to give higher groove ratings to music they were familiar with, but measured no effect of familiarity on groove in the paper’s second study. Witek et al. [5] considered listeners’ familiarity with traditionally groove-related styles, but did not measure an association between familiarity and groove ratings. Stupacher et al. [77], in their second study, investigated whether listeners’ groove ratings were influenced by their familiarity with the music, but did not observe such an influence either. Recently, Madison & Schiölde [82] found that repeated exposure to music (i.e. augmenting listeners’ familiarity with the music) also augmented listeners’ aesthetic appreciation of the music.
- *Proneness to dancing*: Witek et al. [5] showed in 2014 that the variation of syncopation triggered a stronger groove response in listeners who generally enjoyed dancing, compared to other listeners.

This paper presents results from an online listening experiment in which participants assessed the groove quality of 248 popular music drum patterns. These patterns have been reconstructed from Western popular music recordings and represent a cross section of popular music drumming across several decades and styles.

The paper studies the effect of fifteen stimuli- and participant-related predictor variables on the groove ratings. The choice of these variables was based on two ideas concerning the influence of the stimuli’s rhythmic properties on groove:

- *Temporal Regularity*: Clayton [83] and colleagues [84] defined entrainment as an interaction between two or more “oscillators,” which can be understood as periodic (or quasi-periodic) rhythmic processes. Groove research investigates the interaction of two different kinds of rhythmic processes: musical stimuli and listeners’ inner urge to synchronise sensori-motor behaviour in response to the music. In order to qualify as an oscillator (in Clayton’s sense), a musical stimulus must show some degree of temporal regularity. And listeners must be able to detect this regularity in order adapt their sensori-motor behaviour to the music. We hence expect listeners’ ease of detecting temporal regularities to be positively associated with groove (see also [85]). Factors that affect listeners’ perception of temporal regularities might depend on the stimuli (how strongly the regularities are articulated in a stimulus) or on the listeners (their competence to extract regularities from a musical pattern, their familiarity with a repertoire, etc.). Temporal regularity of a stimulus may be a necessary precondition for listeners to entrain their body movement with music. But, by itself, regularity is unlikely to be sufficient to explain the groove phenomenon (if it were, then a isochronous sequence of metronome clicks would represent a high-groove stimulus).
- *Motivation/Interest*: Listeners may also be more or less motivated to entrain their body movements with music. Consequently, factors that increase listeners’ interest in the music and motivate entrainment are likely to be positively associated with groove. These factors, again, may be related to the stimuli (like rhythmic, timbral, harmonic properties of the music that create interest in a listener) or to the listeners themselves (their musical preferences, or their proneness to entrainment in a specific situation).

The music-related factors discussed in previous research either relate to the regularity of the stimuli (*Beat Salience*, *Event Density*) or to musical methods for creating rhythmic interest (*Syncopation*, *Rhythmic Variability*, *Microtiming*).

This study considers the relative relevance of these five factors in one single experiment and investigates a series of further predictor variables that can also be understood to either relate to temporal regularity or rhythmic interest. In addition, we are particularly interested in studying the association of listener-related factors like participants’ *Style Preference*, their *Familiarity* with the repertoire, and their musical *Expertise* with *Groove*. A description of all predictors, their relationship with either temporal regularity, interest/motivation, or both, and our hypotheses how these predictors relate to groove will be given in the *Predictor Variables* section of the *Methods* chapter.

Several of the predictors depend on notated rhythm and cannot be developed on the basis of original audio recordings alone. By reconstructing Western popular music drum patterns based on transcriptions and timing measurements, we intend to make rhythmic aspects more easily accessible.

Using drum patterns as stimuli seems to be reasonable, given that the drum set is considered to be crucial for maintaining meter and tempo in popular music [86–90] and thus for creating temporally regular patterns. Also, the drums create rhythmic interest in popular music [5, 43, 73, 80, 91]. Consequently, they will have an influence on listeners’ motivation to entrain with the music. Accordingly, drum patterns have been used for creating stimuli in previous groove research, either on their own, or in combination with other instruments like the bass [5, 62, 64–69].

This study has an exploratory design insofar as effects have not been systematically varied. Instead, variability depends on the arbitrary sample of drum patterns used in the experiment and on the random sample of participants who took part in the study. Under these circumstances, and also due to strong associations between predictors, we can only roughly estimate the size and nature of the associations between the predictors and groove, but no causal relationships can be established.

## Materials and methods

### Ethics statement

This study collected data about the subjective experience of music listeners in an online survey. The Swiss Federal Law on Research on Humans (Humanforschungsgesetz, HFG, from September 30, 2011) specifies that health-related studies must obtain approval by the regional Ethics Commissions (HFG, Art. 45). Our study is not a health study as defined by the law (HFG, Art. 2) and does therefore not require to be approved by the regional Ethics Commission.

Participants gave informed consent and had the opportunity to give their e-mail-address in case they wanted to be updated about the study’s results or receive invitations to new surveys. E-mail addresses were stored separately from the experimental data in order to guarantee anonymity. No IP addresses were collected during the survey.

### Stimuli

We compiled a list of fifty highly renowned popular music drummers, using the following selection method: drummer names were collected from approximately a dozen different drummer rankings presented on dedicated internet sources (e.g. top 100 or top 50 lists on the *Rolling Stone Magazine*, *Drummer World* websites, or on similar resources). Additionally, we contacted nine professional drummers from our personal networks and from the Lucerne School of Music faculty. These experts belonged to different musical scenes, and they were asked to provide their personal list of the most important drummers in Western popular music.

Approximately 120 different drummers, who have been active between the 1950s and the mid-2010s, were mentioned in more than one list. Two dozen names (like John Bonham, Clyde Stubblefield, Steve Gadd, James Gadson, Questlove and others) appeared on most or even all of the lists; 62 names were mentioned at least five times. These 62 names were further reduced due to practical considerations, mostly connected to the subsequent selection of tracks and excerpts. This resulted in the final selection of 50 drummers who were included in the experiment.

The selection of drummers showed extreme gender bias: only one female drummer was mentioned multiple times in any of the lists (Sheila E., best known for her work with Prince), but her name was mentioned less than five times, so she was not included in the final list. The male predominance in the sample is likely to be connected to an instrument selection bias (boys are much more likely to pick up the drums than girls), deeply rooted in Western society [92–94].

It was not our intention to select the fifty “best” drummers in Western popular music. But we are confident that our selection unites drummers that have an excellent reputation throughout the field. They form a representative sample of highly competent musicians within the genres of rock, funk, R&B, pop, disco, soul, heavy metal and rock’n’roll.

For each of the fifty selected drummers, a list of five tracks was compiled. These tracks were either renowned in the drummer community, because they have a distinctive drum pattern, or/and they had been commercially successful. Tracks recommended by the nine experts and tracks with so-called “iconic” drum patterns (i.e. patterns that are widely taught and studied in drum education) were likely to be chosen. We also consulted chart listings (Billboard Hot 100, UK Singles/Album charts, etc.) and encyclopaedia entries about the drummers, their bands or bandleaders, searching for lists of influential tracks. If drummers had published records under their own name, we chose at least one track from these recordings. These tracks often feature particularly elaborate and non-generic drum patterns. The selection resulted in a list of 250 tracks, which is presented in this paper’s *Supporting Information* section (S1 Table). The selection of tracks represents a wide variety of popular music from mainstream to more experimental music.

From each of the chosen tracks, we selected an excerpt of eight bars. A core criterium for the selection of the passage was that the drummer played a consistent and at least partly repetitive rhythmic pattern (drum solos were excluded from the selection), and that the drum voice was well audible within the sound of the band. Since the tracks have different tempi, the resulting eight-bar selections also differed in duration.

Two researchers, who are also professional jazz musicians (a drummer and a saxophonist), independently transcribed the drum pattern for each of the 250 excerpts using the *Transcribe!* software (version 8.00.3). The transcribers attributed one of four loudness levels to each drum stroke by ear (in the order of diminishing intensity: forte, mezzoforte, piano, ghost note). They revised each others’ transcriptions and discussed differences between the transcriptions until they reached a consensus. The consolidated transcriptions were typeset in *Finale* (version 2014.5).

The timing of each drum stroke onset was measured in *LARA* (version 2.6.3), using spectrograms and oscillograms. The measurement accuracy was additionally checked by ear using *LARA*’s timing marker playback function. Onset measurement is estimated to be accurate to ±3 ms for most of the music excerpts. For a subset of 19 stimuli, onset detection was difficult, because either the drum stroke onsets were hard to identify against the background of the other voices, or the sound quality of the recording was compromised, which mostly occurred in older recordings. But even in these problematic cases, we expect the timing measurement error to rarely exceed ±10ms. The rhythmic patterns with instrumentation, timing, and dynamics data were exported from *LARA* as text files.

The text files were uploaded to a *MySQL* (version 5.5.2.4) database. A MIDI file was created from each of the 250 rhythm patterns. The files represented the timing measurements exactly (the MIDI file settings were adjusted to allow for a time resolution far below the millisecond level). The dynamic information with the four loudness levels was mapped onto MIDI velocity values with a separate mapping rule for each instrument of the drum set such that the resulting audio tracks sounded natural.

Subsequently, the 250 MIDI files were imported into *Avid Pro Tools* (version 12.1). One set of drum sound samples (snare drum, bass drum, toms, hi-hat and other cymbals) was selected from the *Toontrack Superior Drummer* (version 2.4.4) *Custom & Vintage* audio samples library, and all stimuli were reconstructed using these samples. The sampled drum set was chosen to be neutral in the sense that it did not have sound characteristics that are typical for one of the styles, while sounding strange in the context of other styles.

In some patterns, additional percussion instruments (shaker, cowbell, tambourine, cabasa, triangle) play an important role. They were chosen from *Toontrack*’s *Latin Percussion* library and also integrated into the stimuli using *Pro Tools*. Five stimuli featured hand claps that were not available in the *Custom & Vintage* and *Latin Percussion* libraries. The hand claps were recorded by the four researchers during an impromptu recording session in June 2016.

Light reverberation was uniformly added to every audio reconstruction. This created the illusion that all stimuli were played on the same drum set and in the same room acoustics. The goal was to eliminate timbre as a variable from the stimuli as much as possible. The stimuli were exported from *Pro Tools* in mp3 audio file format.

During the experimental run, we noticed that the tambourine had been forgotten in the reconstruction of Steve Gadd’s drum part for Paul Simon’s “50 Ways to Leave Your Lover.” A corrected version of this pattern and an additional reconstruction of another excerpt from the same tune was added into the pool of stimuli, expanding the number of stimuli to a total of 252. The stimuli can be downloaded under www.grooveresearch.ch.

### Procedure

The survey was created on *SoSci Survey* (www.soscisurvey.de), a research platform for online experiments. Participants used a laptop or desktop computer and followed an internet link to start the experiment. All information and texts were presented in Basic English [95] as far as possible.

On the welcome page, participants were informed about the general purpose of the experiment (analysing the impact of rhythm on music perception), about the procedure of the survey, about data collection and anonymisation, and about the scholarly use of the gathered data. Participants then read a declaration of consent and expressed their consent by clicking a button. They answered a series of personal questions concerning their age, country of residence, gender, and musical experience. They indicated their musical taste by expressing their liking for 21 musical styles, using mouse-operated sliders on a 101-point Likert-type scale (ranging from “not at all” to “very much”). Participants were told to leave the slider marker on the “No answer” box (default feedback), if they did not have an opinion on a style or were not familiar with it. The style preference questionnaire was a slightly adapted version of the Short Test of Music Preferences (STOMP) questionnaire [81, 96, 97].

Participants were encouraged to use headphones throughout the experiment. They listened to one stimulus during the trial run and adjusted the loudness to a comfortable level. They completed two simple audiometry tests (counting triangle and bass drum sounds of different loudness) which indicated to the researchers whether the participant’s playback situation and equipment had been adequate for performing the listening test.

Subsequently, participants proceeded to the experimental stimuli: a clip, randomly chosen from the 252 stimuli, was presented to the participant who gave a feedback about her/his experience while listening. Participants rated their agreement with six statements (see *Response Variables* below), also on a 101-point Likert-type scale. By default, all sliders were set to a neutral position (at a medium value of 51), and the extreme positions were labeled (“not at all” and “very much”). Using radio buttons, participants indicated whether or not they thought they knew the band or song featured in the audio clip. And they guessed the musical style/genre of the clip from a genre list (ticking multiple choice boxes). This style list was identical to the list used to assess listeners’ style preferences, except that classical and modern art music were now omitted. When participants had completed their feedback to a stimulus, they were informed about the original recording from which the stimulus had been derived (song title, band name, drummer name, etc.). Subsequently, participants had the option to either continue to the next music example, or to terminate their participation. On the final screen, participants were thanked for their contribution, and they could volunteer to give their e-mail address in order to be sent information about the study’s results or invitations to future surveys.

### Participants

Participants were recruited through several channels: a collective e-mail invitation was sent out to the students and faculty of the Lucerne University of applied Sciences and Arts (approximately 7000 individuals), the researchers invited people from their personal networks through e-mails and social media. Additionally, research managers and department chairs of music departments at Swiss and German universities were contacted and asked to distribute the invitation among their institutions’ students and academic staff. A media information about the project was sent out by the Lucerne University of Applied Sciences and Arts, which was published or mentioned over 50 times in the Swiss and German consumer press (online, print, radio) and in the specialised press (drum magazines).

**Fig 1:**
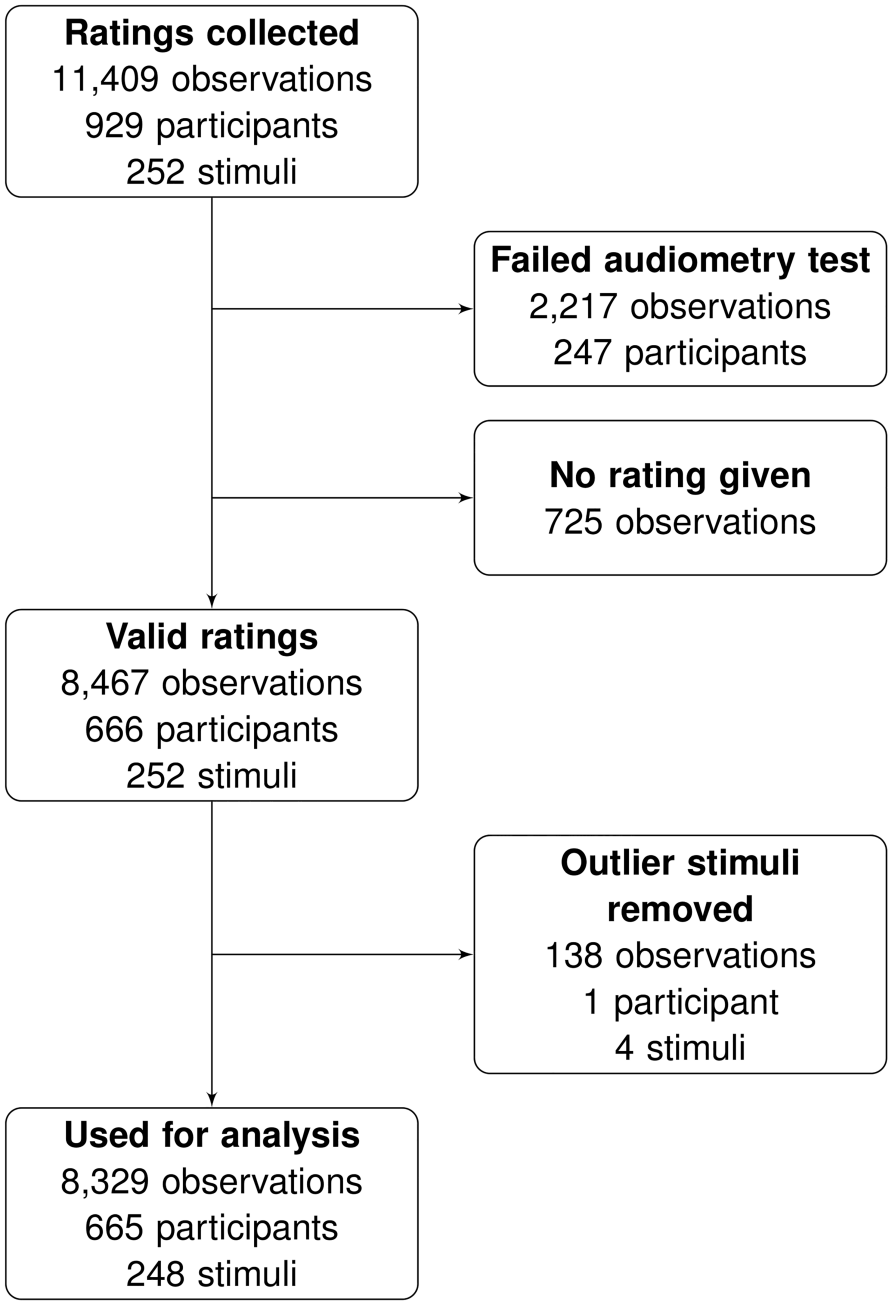
Study flow diagram. Collection and triage of survey data.

These recruiting activities, the media reports in particular, triggered a large number of participations. As can be seen in the flowchart of Fig 1, 929 participants rated the 252 stimuli a total of 11,409 times (a mean of 12.3 stimuli per participant, 8 stimuli in the median). Of those 929 participants, 247 failed the audiometry test (i.e. their event counts were off target by more than 1) and their 2,217 ratings were discarded. 725 observations were discarded because the participants had not displaced any of the rating rulers from their initial medium position, so they had not explicitly expressed an opinion on the stimulus. A total of 8,467 observations with complete rating set, given by 666 participants, were valid.

The mean groove ratings of 4 stimuli were statistical outliers (see *Response Variables* below), and the corresponding observations were removed from the data set. The data set used for analysis consisted of 8,329 observations on 248 stimuli, given by 665 participants. Each of the 248 drum patterns was rated by a mean of 33.6 participants.

Of the 665 participants, 145 were female, 511 male, 1 self-identified as a different gender, and 8 refused to answer. Participants had an age range between 18 and 77 years ($\bar{x}=40.5$, *s* = 13.7). 194 of the participants declared to be professional musicians; 280 self-identified as amateur musicians; 179 stated to be music listeners. 317 participants stated that Switzerland was their country of residence, 279 indicated living in Germany, 51 individuals participated from other, predominantly European countries.

The composition of the participants group shows considerable self-selecting sample bias [98, 99] with respect to two characteristics: male participants and people with high musical competence are overrepresented in the sample, compared to a general central European population. The high proportion of male subjects may again be traced back to gender bias in music education and practice: the drum set is predominantly played by male musicians and music students [92–94], so men could be expected to be more interested in the survey than women. The high proportion of musically competent subjects is most probably associated with two recruitment-related circumstances: firstly, e-mail-based recruitment was focused on academic institutions dedicated to music performance or research (music departments, conservatoires). We expected a high turn-out from these institutions, because their faculty and students are likely to be interested in the experiment. Secondly, three drum magazines published invitations to participate in the experiment (print and online); these magazines target professional and amateur drummers and hence have a musically competent readership. It is likely that the open-ended design of the experiment further accentuated the bias: we may expect participants with high interest in popular music and in the drums to muster more patience for carrying out the experiment, and to rate more stimuli than a person with less interest. The sample predominantly includes participants from Switzerland and Germany, due to the fact that the media information was circulated in these two countries and in German language only.

**Fig 2:**
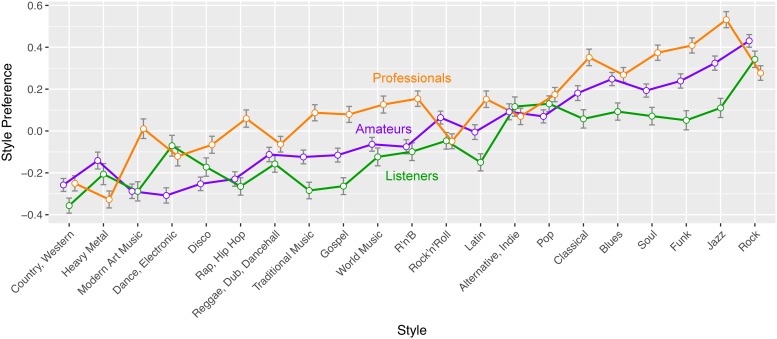
Style preference. Participants’ mean *Style Preference* ratings (*z*-scores) for 21 styles, grouped by musical *Expertise*. Error bars show the standard error of the mean.

Participants’ mean style preferences are presented in Fig 2, split by musical *Expertise*. Rock, jazz and funk were most popular within this sample of participants, whereas country/western, heavy metal and modern art music were least appreciated. Music *professionals*’ love for music showed in their generally high appreciation for a wide range of musical styles. Compared to the other groups, they offered a relatively high approval for styles that are taught at music schools and conservatoires, like jazz, classical and modern art music, but also for styles with an ethnic background (like latin, gospel, world music, traditional music). *Amateurs* and *listeners* on the other hand had a high opinion of rock and rock-related styles, compared to the *professionals*.

### Response variables

Upon listening to a reconstructed drum pattern, participants used sliders to indicate their agreement with six statements related to the groove experience (Table 1).

**Table 1:** Questionnaire response items with inter-item correlations and factor loadings.

Item	Statement	———Inter-Item Correlations———						Factor Loadings
		S1	S2	S3	S4	S5	S6	
S1	I would like to dance to this music.							0.87
S2	I like to listen to this music.	0.64						0.69
S3	This music is great for a party.	0.70	0.56					0.75
S4	The beat of this music is easy to recognise.	0.18	0.08	0.26				
S5	This music has an interesting rhythm.	0.37	0.63	0.29	−0.15			
S6	Something in this music is disturbing.	−0.26	−0.35	−0.29	−0.17	−0.12		
**Groove** (first Principal Component of S1, S2, S3)		0.91	0.82	0.86	0.20	0.48	−0.33	

The first item, S1 (“I would like to dance to this music.”), prompts participants to comment on their entrainment response while listening to a stimulus. With response S2 (“I like to listen to this music.”) participants signalled their pleasure or enjoyment while listening. Finally, response S3 (“This music is great for a party.”) invokes a social situation which is frequently associated with both dancing/entrainment and pleasure. Entrainment and pleasure are the core components of the canonic definition of groove in music psychology [4, 5].

A preliminary parallel analysis [100] suggested to extract one factor from response variables S1–S3 (Kayser’s criterion). An exploratory factor analysis was carried out; the factor loadings for S1–S3 can be studied in Table 1. The analysis showed that the three variables reliably (Cronbach’s *α* = 0.81) load on one factor that represents 60% of the variance in the three variables. The *Groove* scale is defined as the first principal component of items S1, S2, and S3. It estimates the strength of participants’ groove experience and represents the primary outcome variable used in the analysis. The distribution of the *Groove* ratings was fairly symmetric (skewness *γ*_1_ = −0.263) and not excessively platykurtic (kurtosis *γ*_2_ = −0.427). As expected, the *Groove* scale was strongly correlated with S1, S2, and S3 (Table 1).

A side note: by operationalising entrainment with item S1 in the questionnaire, we make the implicit assumption that people generally like to express entrainment in dance. But this is not necessarily true: some people might not be inclined to dancing [5], but they might nevertheless feel the urge to move along with music. For these people, the wording of the questionnaire potentially introduces negative bias into the *Groove* ratings. However, the high factor loading of the S1 item and the overall good reliability of the composite *Groove* scale does not indicate that this kind of bias substantially affected the collected data.

The *Beat Recognition* variable consists of participants’ responses to item S4 (“The beat of this music is easy to recognise”), in which they subjectively assessed how easily they captured the beat in the stimuli. This variable operationalises participants’ ease of perceiving temporal regularities in the stimuli, outlined as a precondition for entrainment in the *Introduction*.

The Pearson correlation between *Beat Recognition* and *Groove* was positive, as we would expect, but surprisingly weak (*r* = 0.20, *p* < 0.001). Potentially, this weak association was a consequence of participants’ general familiarity with the stimuli. Most of the participants lived in central European countries. We may expect them to know the Western popular music repertoire and to easily detect the beat. The empirical data seems to confirm this: the *Beat Recognition* ratings were negatively skewed (*γ*_1_ = −0.525), indicating that the participants generally had little trouble finding the beat in the stimuli. Thus, the necessary precondition for entrainment was satisfied most of the time.

The *Rhythmic Interest* variable consists of participants’ responses to item S5 (“This music has an interesting rhythm”), in which they indicated the interest they experienced while listening to a stimulus. This variable operationalises the interest/motivation topic from the *Introduction*. As expected, *Rhythmic Interest* was positively correlated with *Groove* (*r* = 0.48, *p* < 0.001). This supports the notion from the *Introduction* that interest might create motivation in listeners to entrain to music. *Rhythmic Interest* was most strongly correlated with S2 (“I like to listen to this music”), the component of the *Groove* scale that is most related to pleasure (*r* = 0.63, *p* < 0.001). This makes sense in the light of recent neuro-scientific research, which found a close connection between pleasure and interest in aesthetic experiences [101].

The responses to item S6 (“Something in this music is disturbing.”), finally, form a control variable we called *Disturbance*. This variable registered when participants became irritated by some aspect of a stimulus, and it can be understood as an indicator for poor stimulus reconstruction. The ratings of four stimuli (138 observations, see Fig 1) were excluded from the analysis, because they were statistical outliers: they had very low mean *Groove* ratings, and their mean S6 ratings were high.

The four outcome variables (*Groove*, *Beat Recognition*, *Rhythmic Interest*, *Disturbance*) of the remaining 8,329 observations by 665 participants on 248 stimuli were *z*-standardised, i.e. centralised to a mean of 0 and scaled to a variance of 1.

### Predictor variables

The selection of this study’s fifteen predictor variables was guided by the two theoretical arguments outlined in the *Introduction*, namely that listeners’ experience of groove might be influenced by their perception of temporal regularity (which arguably is associated with their capability of entraining their body movement with the music) and by the rhythmic interest the music creates in them.

#### Tempo, tempo change, and tempo instability

Three tempo-related variables were calculated using quadratic regression models, which relate physical onset times (in seconds) and metrical onset times (in beats) for each stimulus. The initial *Tempo* (in bpm) of a stimulus was derived from the model’s linear coefficient.

Previous research showed that medium tempi around 100 bpm are particularly well suited for entrained behaviour like walking to music [102–104]. Listeners’ might be particularly motivated to move along with music, if the beat rate can easily be mapped onto periodic body motion, so we would expect medium tempi to be best for *Groove*. This hypothesis has been confirmed in recent research by Etani et al. [79]. However, motion capture studies on body movement in response to music have also shown that different body parts entrain to different tempi or metric levels [105, 106]. This suggests that the relationship between *Tempo* and *Groove* has several facets: a good tempo for finger tapping might be too fast for the periodic movement of larger and heavier body parts, like bobbing the head or swaying the torso.

*Tempo Change* was derived from the tempo model’s quadratic term: this variable indicates how much the tempo slows down (negative values) or accelerates (positive values) across the stimulus. It is measured in beats per square minute (or bpm^2^). *Tempo Instability* is defined as the absolute value of *Tempo Change*: *Tempo Instability* increases with both stronger accelerando or ritardando. We expect higher *Tempo Instability* to decrease listeners’ sense of regularity in a stimulus, and we hypothesise that *Tempo Instability* is negatively associated with *Groove*.

#### 8^th^ note swing, 16^th^ note swing, and residual microtiming

Three measures of microtiming magnitudes were calculated for each stimulus: *8^th^ Note Swing* refers to the swing ratio on the level of the eighth note, which stands for the mean ratio between the durations of the onbeat and offbeat eighth notes [48, 107]. This value was calculated for a stimulus only if its pattern contained a total of at least 16 offbeat eighth notes, which was the case for 241 of the 248 stimuli. *16^th^ Note Swing* is a similar measure for the swing ratio between sixteenth notes. It was calculated if there were at least 32 offbeat sixteenth notes in the whole pattern, which was the case for 146 stimuli. Rhythms with greater swing ratios (i.e. larger inequality between the longer first and the shorter second note) are thought to improve the perception of the beat [108] and, consequently, to strengthen the perception of temporal regularities. We hypothesise that the two swing measures are positively associated with *Groove*.

In order to calculate the *Residual Microtiming* magnitude, a grid of expected time positions was calculated using the quadratic tempo model (see *Tempo* and *Tempo Change* above) and the *8^th^ Note* and *16^th^ Note Swing Ratios* (if applicable). For each stimulus, the *Residual Microtiming* variable represents the density-adjusted standard timing deviation (see [42]) of the residuals after *Tempo*, *Tempo Change*, and the two swing ratios were accounted for. The interpretation of *Residual Microtiming* is similar to the interpretation of unsystematic microtiming proposed by Hellmer & Madison [61]. According to Merker [85], *Residual Microtiming* is likely to confuse the perception of temporal regularity in listeners, and we expect it to be negatively associated with *Groove*.

#### Beat salience and event density

Our method to measure *Beat Salience* was derived from the method described by Madison et al. [3]. It is based on calculating the autocorrelation function (ACF) of a stimulus’ preprocessed audio signal and retrieving the value of the ACF for the time lag corresponding to the beat duration. Note that the beat duration equals a quarter note in all 248 stimuli. *Beat Salience* is a measure of how well the beat is audible in a stimulus, and we expect it to relate quite directly to listeners’ perception of temporal regularity. Consequently, we hypothesise that *Beat Salience* is positively associated with *Groove*, in line with previous research [3]: the more salient the beat, the stronger listeners’ perception of temporal regularity.

*Event Density* is the average number of drum strokes per beat. The number of strokes in a stimulus was counted on the basis of the transcription. High *Event Density* is likely to increase both listeners’ notion of temporal regularity (because listeners frequently obtain metric and rhythmic information) and the rhythmic interest created by the pattern [42, 109]. Thus, we expect *Event Density* to be positively associated with *Groove*.

#### Syncopation

*Syncopation* was measured using a slightly modified version of the method presented by Witek et al. [5], which itself is based on a procedure developed by Longuet-Higgins and Lee [70]. Witek et al.’s method (see Text S2 in the Supporting Information section of [5] and a corrected version in [74]) attaches a numeric syncopation value to each stroke in a drum pattern and sums these values up in order to obtain an overall syncopation measure. The contribution of each event to syncopation depends on the event’s position in the meter, on whether it precedes a rest on a metric position with greater weight, and on rules dealing with the multi-layered (or polyphonic) nature of popular music drum patterns. Witek et al.’s method allows to measure syncopation in the bass drum and snare drum. Syncopation in the hi-hat is not covered by their method, because the hi-hat rhythm was not varied in their experiment.

The contributions of the bass drum and the snare drum to *Syncopation* was carried out according to the rules of Witek et al. [5]. We made only three minor adjustments to Witek et al.’s procedure: we added a simple rule to accommodate triplets; we treated the toms analogous to the snare drum; and we averaged the syncopation values over the 32 beats (or quarter notes) of a pattern. The modeling resulted in the *Syncopation* variable, which is given as mean syncopation per beat and can be used independently from pattern length.

**Fig 3:**
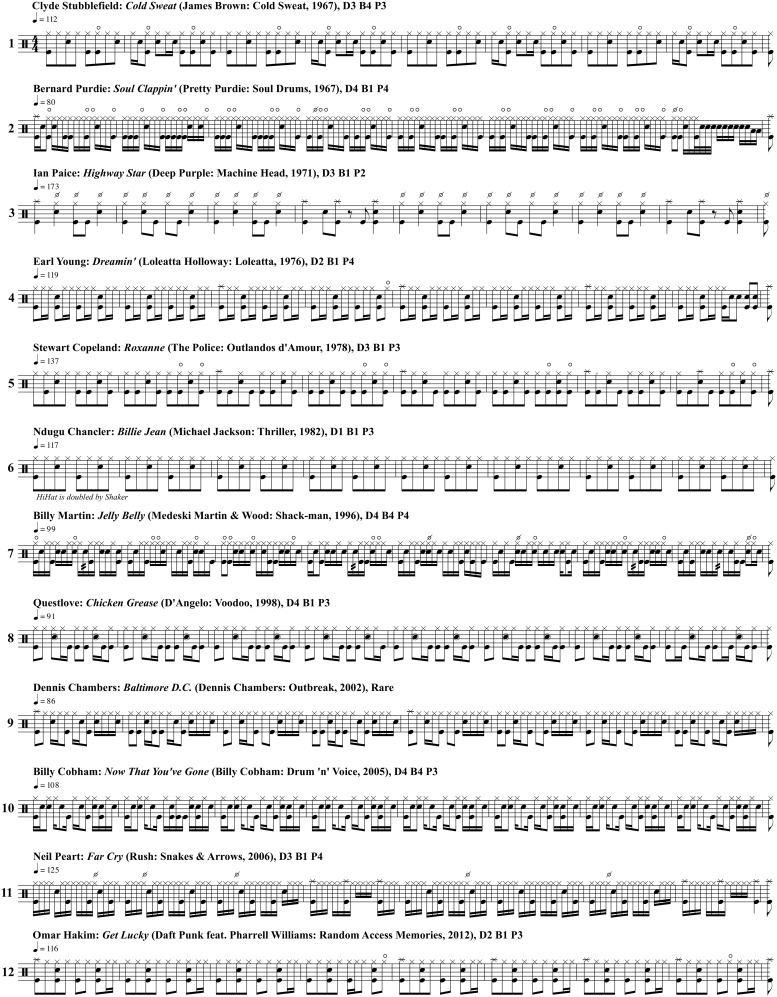
Stimuli. Transcription of one typical example for each *Pattern Category*.

The cymbals (hi-hat, ride cymbal, crash cymbal) were not considered for the calculation of *Syncopation*, even though these voices were rhythmically varied in this study’s stimuli. Losing the contribution of the hi-hat to the syncopation variable is regrettable, but it can be justified to a certain extent: as can be seen in the transcriptions of Fig 3, the hi-hat plays regular rhythmic patterns most of the time. We expect snare drum and bass drum rhythms to be more relevant for the creation of syncopation than rhythms in the hi-hat or ride cymbal. Further, the main reason for rhythmic variability in the hi-hat are different playing techniques (closed, half-open, open hi-hat) and dynamics rather than the sequence of played notes and rests. To estimate the contribution of these heterogenous textures to the overall syncopation value would require a thoroughly new approach to syncopation measurement, which was not attempted in this study.

*Syncopation* is an interesting factor with respect to the temporal regularity and interest/motivation topics: on one hand, we can expect *Syncopation* to add rhythmic interest to a drum pattern and therefore increase listeners’ motivation to move along with the music. On the other hand, we expect *Syncopation* to challenge listeners’ sense of the beat by definition and to undermine their perception of temporal regularity. Witek et al.’s idea that the relationship between *Syncopation* and *Groove* potentially follows a ∩-shaped Wundt curve may be understood as a conflicting influence of temporal regularity and interest. Patterns with low *Syncopation* show clear temporal regularity but may be boring to the listener, whereas patterns with high *Syncopation* are more interesting, but may confuse listeners’ sense of regularity and meter.

#### Rhythmic periodicity and rhythmic variability

The rhythmic information of each pattern given in the transcription was stored in form of a matrix. Dimensions of the matrix coded the different instruments of the drum set, and all possible metric positions to the level of the 16^th^ triplet and binary 32^nd^ notes. A stroke on a drum or cymbal was coded in the matrix as a “1”, whereas an empty position was coded as “0”. The matrix was reshaped in several ways to form three-dimensional arrays with the third dimension representing sub-patterns of one, two, or four bars length. Distance measurements (Jaccard distance [110]) between the sub-patterns of different length were used to classify each pattern as a 1-bar, 2-bar, or 4-bar pattern: each stimulus was assigned to the sub-pattern category that resulted in the smallest mean Jaccard distance. This classification entered the analysis as the categorial *Rhythmic Periodicity* variable with three levels (1-bar, 2-bar, 4-bar sub-patterns). We expect patterns with a shorter period to have greater temporal regularity than patterns with a longer period. Conversely, patterns with a longer period might be considered to be more interesting than patterns with a shorter period.

The corresponding mean Jaccard distance among sub-phrases of the chosen *Rhythmic Periodicity* category was additionally stored for each pattern as a measure of *Rhythmic Variability*: high mean distances indicate that the pattern changes a lot between the iterations of the one-, two-, or four-bar sub-patterns. A mean distance of zero means that the sub-pattern repeats identically in each iteration. Patterns with high *Rhythmic Variability* can be expected to show less temporal regularity and create more rhythmic interest than patterns with low *Rhythmic Variability*.

We do not have a clear expectation how the two predictors *Rhythmic Periodicity* and *Rhythmic Variability* relate to *Groove*.

#### Expertise, familiarity, and style preference

We tested three participant-related variables for their associations with *Groove*. Participants’ self-identification with either the *professional* musician, *amateur* musician, or *listener* groups constituted an *Expertise* predictor variable (the few data points from the *not interested* and *no information* groups were discarded for this analysis). We expect the *professional* musicians to have greater ease detecting temporal regularities in a stimulus compared to the *amateur* musicians, who in turn can be expected to detect regularities more easily than the *listeners*. Conversely, the *professional* and *amateur* musicians might more easily get bored with a simple rhythm, compared to the *listeners*.

After listening to a stimulus, participants answered the question “Do you think that you might know the band or the piece?” by ticking yes/no boxes. This resulted in the binary *Familiarity* predictor variable (*familiar*/*unfamiliar*). Participants were not asked to prove their familiarity by writing down the song title or the band. We expect participants to be more motivated to entrain with a stimulus, if they have the impression to know the music (see also [82]).

A third participant-related variable is *Style Bias*, which represents how much a participant likes the style she/he thinks a drum pattern belongs to. This variable combines information from different parts of the survey: in the early stage of the experiment, participants rated their preferences with respect to 21 musical styles (see Fig 2). In the later stage of the experiment, participants listened to a stimulus, provided *Groove* ratings, and chose one or more styles that they thought the drum pattern was associated with. For each observation, the *Style Bias* variable takes the value of the participants’ preference for the style that she or he thinks the stimulus belongs to. If the participant indicated that the stimulus might belong to two or more styles, the *Style Bias* variable took the participants’ mean preference for the selected styles. We expect participants to be more motivated to entrain with music from a style they like.

#### Pattern category

*Pattern Category* is a stimulus-related predictor variable that classifies the patterns into groups with similar rhythmic features. To our knowledge, a variable based on structural aspects of a notated drum pattern (besides *Syncopation*) is a novelty in groove research. Accordingly, we will take some care describing the definition of this variable and its categories in sufficient detail.

At its core, a Western popular music drum pattern usually consists of three rhythmic layers:

- The *Downbeat* layer normally features the bass drum [88, 89]. In many cases, the primary and/or secondary downbeats (first and third beats of the common time bar) are played as part of the rhythm in this layer.
- The *Backbeat* layer is often played on the snare drum [87, 89], and it frequently plays one or both of the backbeats (beats two and four of the bar).
- The *Pulse* layer is usually played on the hi-hat cymbals or the ride cymbal [87, 89, 90]. It often presents a (more or less regular) sequence or pulsation of notes. In most patterns, the pulsation is faster than the quarter note beat.

These three core layers may be complemented by other elements: in many patterns, the drummer plays fills (virtuoso gestures often played on tom drums) and crashed cymbal strokes to mark the end of larger formal units (mostly 4-bar or 8-bar units). Additional percussion instruments may lend a special flavour to a pattern. Sometimes, these percussion instruments are played by additional musicians, whereas the fills and cymbal accents are played by the drumset player himself.

The *Pattern Category* variable classifies the drum patterns according to the properties of the three core rhythmic layers using a semi-automatic, iterative classification method that is based on calculating the proportion of off-beat positions played in the respective layer. As a result, each pattern is assigned to one category in each layer.

There are four options for the categorisation of a pattern’s *Downbeat* layer: the *D1 generic* category unites patterns, in which the bass drum quite exclusively plays the two downbeats of the bar (beats 1 and 3), and nothing else. In the *D2 four to the floor* category, the bass drum plays on all four beats. Patterns in the *D3 eighth* and *D4 sixteenth* categories show a substantial proportion of off-beat eighth and sixteenth notes, respectively. A few patterns did not meet the threshold of any category. They ended up in a catch-all category that will not be used in the analysis. The categories can broadly be defined by the metric grid they require in notation: patterns in the *D2 four to the floor* category can be notated with quarter notes only; for those in the *D3 eighth* category, an eighth note grid must be used; and patterns in the *D4 sixteenth* category require a sixteenth note level.

The categorisation procedure was similarly and independently carried out for the *Backbeat* layer: in the *B1 generic* category, both backbeats (on 2 and 4) are played, but not much else. Denser rhythmic snare drum patterns were assigned to the *B2 quarter*, *B3 eighth*, and *B4 sixteenth* categories. Finally, there were three categories for the *Pulse* layer: *P2 quarter*, *P3 eighth*, *P4 sixteenth*.

In theory, there are 48 possible feature combinations, see Table 2. But only eleven feature combinations were frequent among the 248 patterns and were represented by at least five patterns. The number of patterns for each group is printed in bold numbers in the table. Together, the eleven groups represent 200 of the 248 patterns. Stimuli in which the proportion of eighth triplets or sixteenth triplets in any of the layers exceeded a certain threshold were also assigned to the catch-all categories and do not show up in the table.

**Table 2:** Number of stimuli per *Downbeat*/*Backbeat*/*Pulse* feature combination.

Downbeat	Backbeat	Pulse		
		P2 quarter	P3 eighth	P4 sixteenth
D1 generic	B1 generic	1	**10**	1
	B2 quarter	–	–	–
	B3 eighth	–	1	–
	B4 sixteenth	1	2	1
D2 four to the floor	B1 generic	–	**13**	**5**
	B2 quarter	–	1	–
	B3 eighth	–	2	–
	B4 sixteenth	–	1	–
D3 eighth	B1 generic	**10**	**44**	**6**
	B2 quarter	–	2	–
	B3 eighth	1	4	1
	B4 sixteenth	1	**19**	3
D4 sixteenth	B1 generic	2	**24**	**21**
	B2 quarter	–	2	–
	B3 eighth	–	1	4
	B4 sixteenth	1	**30**	**18**

*Note*: Cells with *n* ≥ 5 stimuli in bold print. Number of patterns in the catch-all categories are omitted.

We defined these eleven large groups to be the eleven categories of the *Pattern Category* variable, each representing a specific combination of *Downbeat*, *Backbeat*, and *Pulse* features. Fig 3 shows the transcription of one exemplary drum pattern for each of the categories. For example, Ndugu Chancler’s drum pattern for Michael Jackson’s 1982 hit “Billie Jean” (No. 6 in Fig 3) is a representant of the “archetypical rock pattern” [87]. It shows a generic *Downbeat* in the bass drum with strokes on beats 1 and 3 (*D1*). The “Billie Jean” pattern also features a generic *Backbeat* with snare drum strokes on beats 2 and 4 of the beat (*B1*), and an eighth-note based *Pulse* in the hi-hat (*P2*). The transcriptions of Fig 3 represent the patterns in standardised drumset notation [111]: the bass drum is notated on the bottom line of the staff, the snare drum above the middle line, and the hi-hat above the top line (with crosses as noteheads).

**Fig 4:**
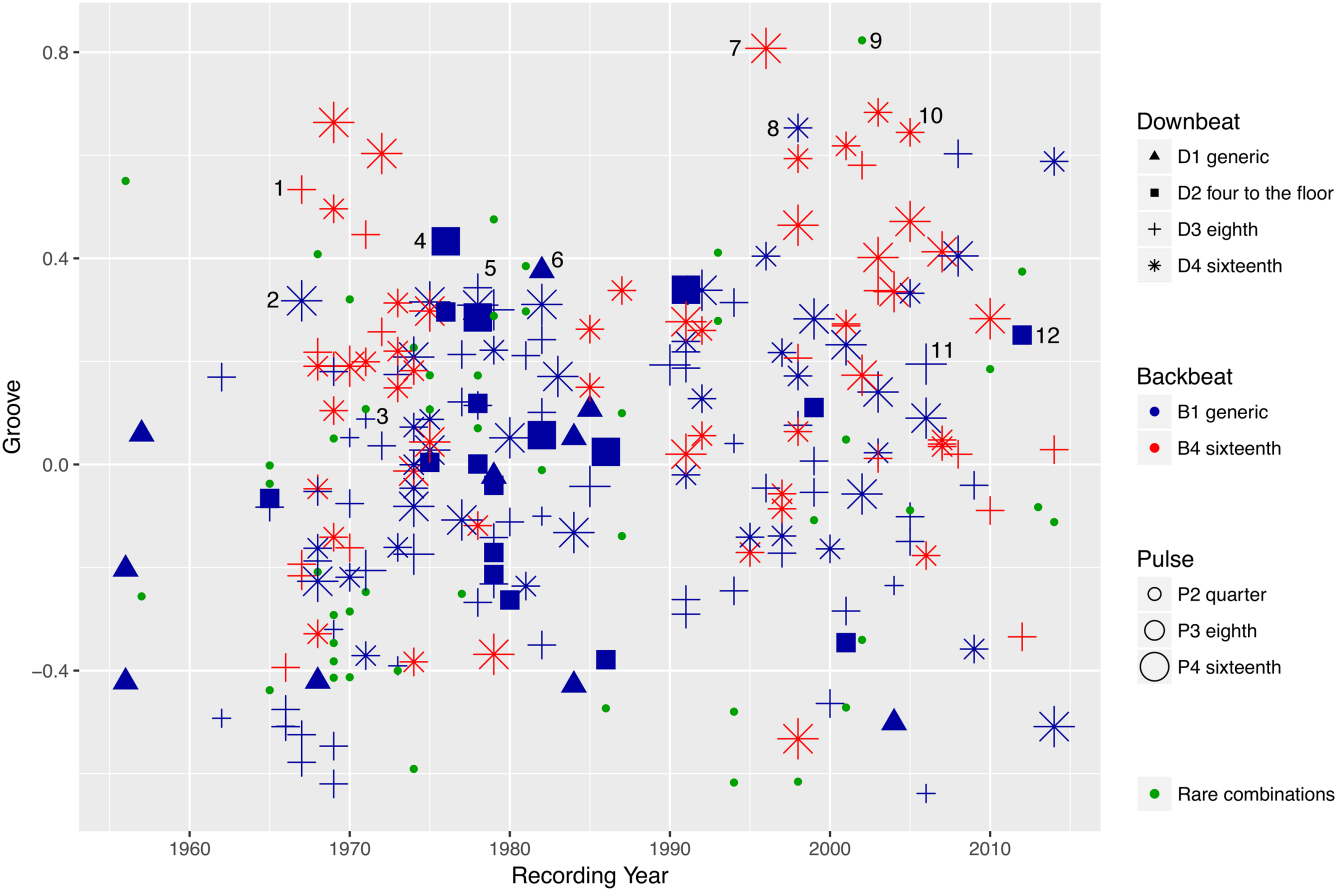
Mean groove rating vs. recording year. Mean *Groove* ratings of all 248 stimuli as a function of *Recording Year*. Number keys are given in Table 3.

Fig 4 shows the mean *Groove* ratings for all 248 stimuli as a function of their recording year. Each symbol shape encodes the *Downbeat* category, while the symbol colour denotes the *Backbeat* layer, and the symbol size represents the category of the *Pulse*. The “archetypical rock pattern,” for example, is represented by medium-sized blue triangles. It can be seen that, in our sample, it shows up frequently during the rock’n’roll era of the 1950s up to the 1980s.

**Table 3:** Typical examples for each *Pattern Category*.

No.	Year	Groove	Drummer	Track	Act	Category
1	1967	0.533	Clyde Stubblefield	Cold Sweat	James Brown	D3 B4 P3
2	1967	0.318	Bernard Purdie	Soul Clappin’	Pretty Purdie	D4 B1 P4
3	1971	0.088	Ian Paice	Highway Star	Deep Purple	D3 B1 P2
4	1976	0.433	Earl Young	Dreamin’	Loleatta Holloway	D2 B1 P4
5	1978	0.343	Stewart Copeland	Roxanne	The Police	D3 B1 P3
6	1982	0.376	Leon Ndugu Chancler	Billie Jean	Michael Jackson	D1 B1 P3
7	1996	0.808	Billy Martin	Jelly Belly	Medeski Martin & Wood	D4 B4 P4
8	1998	0.653	Questlove	Chicken Grease	D’Angelo	D4 B1 P3
9	2002	0.823	Dennis Chambers	Baltimore D.C.	Dennis Chambers	Rare
10	2005	0.644	Billy Cobham	Now That You’ve Gone	Billy Cobham	D4 B4 P3
11	2006	0.195	Neil Peart	Far Cry	Rush	D3 B1 P4
12	2012	0.251	Omar Hakim	Get Lucky	Daft Punk / Pharrell Williams	D2 B1 P3

Each of the eleven pattern categories is loosely associated with specific styles: the blue crosses, for example, stand for patterns with a generic *Backbeat* on beats 2 and 4 (*B1*), and eighth note patterns in the *Downbeat* layer (*D3*). These patterns are frequently associated with rock: examples are Ian Paice’s drum pattern on Deep Purple’s “Highway Star” (No. 3, 1971), Stewart Copeland’s pattern on the Police hit “Roxanne” (No. 5, 1978), or Neil Peart’s pattern on Rush’s “Far Cry” (No. 11, 2006).

A complex, syncopated *Backbeat* layer based on a sixteenth note grid (*B4*) is typical for funk drum patterns; these patterns are represented as red symbols. Clyde Stubblefield’s pattern on James Brown’s “Cold Sweat” (No. 1, 1967) shows this kind of *Backbeat*, but also Billy Martin’s highly elaborate and dense pattern on “Jelly Belly” (No. 7, 1996), or Billy Cobham’s drum pattern on “Now That You’ve Gone” (No. 10, 2005). As can be seen in Fig 4, these patterns have their first heyday in the golden era of funk in the 1960s and 1970s, but they remain popular among funk and fusion drummers until today.

Patterns with generic *Backbeats* (*B1*) and a bassdrum kick on every quarter note (*D2*, also called a “four to the floor” bass drum pattern) are typical for disco, and they are represented in Fig 4 by blue squares. Fig 4 shows that these patterns were particularly popular in the disco era of the 1970s and 1980s. One example from this time is Earl Young’s drum pattern on Loleatta Holloway’s “Dreamin’” (No. 4, 1976). A more recent example is Omar Hakim’s pattern on Daft Punk’s “Get Lucky” (No. 12, 2012). Today, four to the floor bass drum patterns are frequent in electronic dance music.

Patterns with a complex sixteenth-note-based *Downbeat* layer (*D4*) but a generic *Backbeat* (*B1*) are often associated with rap or hip hop (blue stars). A typical example is Questlove’s drum pattern for D’Angelo’s “Chicken Grease” (No. 8, 1998). However, funk/soul tracks may also feature this kind of drum pattern, like Bernard Purdie’s pattern on “Soul Clappin’” (No. 2, 1967).

The eleven *Pattern Categories* with a total of 200 stimuli were used as a categorial predictor for *Groove*. Note that the remaining 48 stimuli are represented in Fig 4 as green dots, but they do not constitute a category on their own. These patterns with rare *Downbeat*/*Backbeat*/*Pulse* combinations span the whole post-war era until today, and the whole range of *Groove* ratings. In fact, the pattern with nominally the highest *Groove* ratings throughout the entire experiment (Dennis Chambers’ drum pattern on “Baltimore D.C.,” No. 9) belongs to one of these rare combinations.

We expect *Pattern Categories* that allow for fine subdivisions of the beat to offer opportunities for original and/or complex rhythm. We anticipate that, in general, patterns with sixteenth note subdivisions in one or more of the layers are rhythmically interesting and therefore obtain high *Groove* ratings. Conversely, we expect participants to more easily perceive temporal regularities when listening to stimuli with generic *Downbeats* and *Backbeats*, which in turn might also affect the *Groove* ratings positively.

### Statistical analysis

The statistical analyses were carried out using *R* (version 3.3.1), and *RStudio* (version 1.0.136). Data visualisations and plots were created with the *R* library *ggplot2* (version 2.2.1). The significance level was set to *α* = 0.05.

The analysis offers an overview of the relative effects of the fifteen predictor variables. Each predictor is primarily studied on its own, and its isolated effect on *Groove* is estimated. There will be no attempt at creating one comprehensive model that integrates all relevant effects. Such an attempt would be futile due to a number of problems: some predictors only have valid values for a subset of the observations, and group sizes are unbalanced. In some cases, however, the joint effect of two or more variables is analysed in order to investigate interactions and dependencies between predictors. Only significant interactions will be reported in the *Results* section.

Effects of categorial variables are measured using analysis of variance, and corresponding effect sizes are given as *η*^2^. Group sizes are almost always unbalanced, but according to [112], a lack of balance does not affect the results of single-factor analysis of variance in a relevant way. Effects of continuous predictor variables are measured using regression analysis, and their size is expressed as *R*^2^. Following rules of thumb formulated by Cohen [113] and Miles & Shevlin [114], we consider effects of *η*^2^ = 0.01 to be small, *η*^2^ = 0.06 represents a medium effect, and effects of *η*^2^ = 0.14 and greater are large. Note that both, *η*^2^ and *R*^2^, give the effect size as a proportion of explained variance and are hence comparable with each other.

## Results

The effect sizes given in Table 4 estimate the variance in the *Groove* ratings that are explained by the fifteen predictor variables. No statistically significant effect on *Groove* was measured for *Beat Salience*, *Residual Microtiming* or *Pattern Variability*.

**Table 4:** Stimuli- and participant-related effects on *Groove*.

Effect	p	Measure	Size	r
Style Bias × Familiarity	<0.001	*R*^2^	0.152	
Style Bias	<0.001	*R*^2^	0.123	+ 0.351
Stimuli	<0.001	*η*^2^	0.096	
Familiarity	<0.001	*η*^2^	0.051	
Pattern Category × Expertise	<0.001	*η*^2^	0.031	
Syncopation × Event Density × Expertise	<0.001	*R*^2^	0.020	
Pattern Category	<0.001	*η*^2^	0.018	
Event Density × Expertise	<0.001	*R*^2^	0.016	
Syncopation × Expertise	<0.001	*R*^2^	0.016	
Event Density × Syncopation	<0.001	*R*^2^	0.013	
Event Density	<0.001	*R*^2^	0.011	+ 0.104
Syncopation	<0.001	*R*^2^	0.010	+ 0.100
Tempo Instability	<0.001	*R*^2^	0.004	−0.063
8^th^ Note Swing	<0.001	*R*^2^	0.004	−0.063
16^th^ Note Swing	<0.001	*R*^2^	0.004	+ 0.062
Expertise	<0.001	*η*^2^	0.003	
Tempo	<0.001	*R*^2^	0.002	−0.044
Tempo Change	<0.001	*R*^2^	0.002	−0.040
Pattern Periodicity	0.288		—	
Pattern Variability	0.338		—	
Residual Microtiming	0.482		—	
Beat Salience	0.804		—	

*Notes*: *p*: significance probability; *r*: Pearson correlation coefficient.

Effects of *Tempo Change*, *Tempo*, *Expertise*, *16^th^ Note Swing*, *8^th^ Note Swing* and *Tempo Instability* were statistically significant, but tiny. *Tempo Instability* seems to be negatively associated with *Groove*, indicating that participants had a slight tendency to give higher *Groove* ratings to stimuli with stable tempo, compared to stimuli with either accelerating or decelerating tempo. Also, *16^th^ Note Swing* appears to be positively related with *Groove*, whereas *8^th^ Note Swing* was negatively associated with *Groove*: participants indicated that a stronger shuffle on the sixteenth-note level was weakly positive for *Groove*, whereas the shuffle on the eighth-note level was negative.

The association between *Tempo* and *Groove* was minuscule and negative. We observed no convex, curved relationship suggesting the existence of an optimal tempo for groove (see Etani et al. [79]). However, we can assume that the drummers chose their patterns in function of the song and of its tempo, so that all tempi can be considered to be optimal within their contexts.

### Syncopation and event density

Across the sample of stimuli, the distribution of *Syncopation* measurements was positively skewed (*γ*_1_ = 0.924). The distribution became approximately symmetric (*γ*_1_ = 0.153) after a square root power transformation. *Syncopation* was positively correlated with *Rhythmic Interest* (*r* = 0.381) and negatively with *Beat Recognition* (*r* = −0.168). This supports our assumption that *Syncopation* makes a drum pattern more interesting to the listeners, but it makes it more difficult to keep track of the beat.

Similarly, *Event Density* was positively skewed (*γ*_1_ = 0.656). This distribution was made more symmetric using a log-transformation prior to the analysis (*γ*_1_ = 0.039). *Event Density* was positively correlated with *Rhythmic Interest* (*r* = 0.302), but it was unrelated to *Beat Recognition* (*r* = −0.080). This confirms our assumption that a more dense rhythm is likely to be perceived as more interesting. However, unlike assumed earlier, rhythmic density does not seem to affect listeners’ beat recognition.

**Fig 5:**
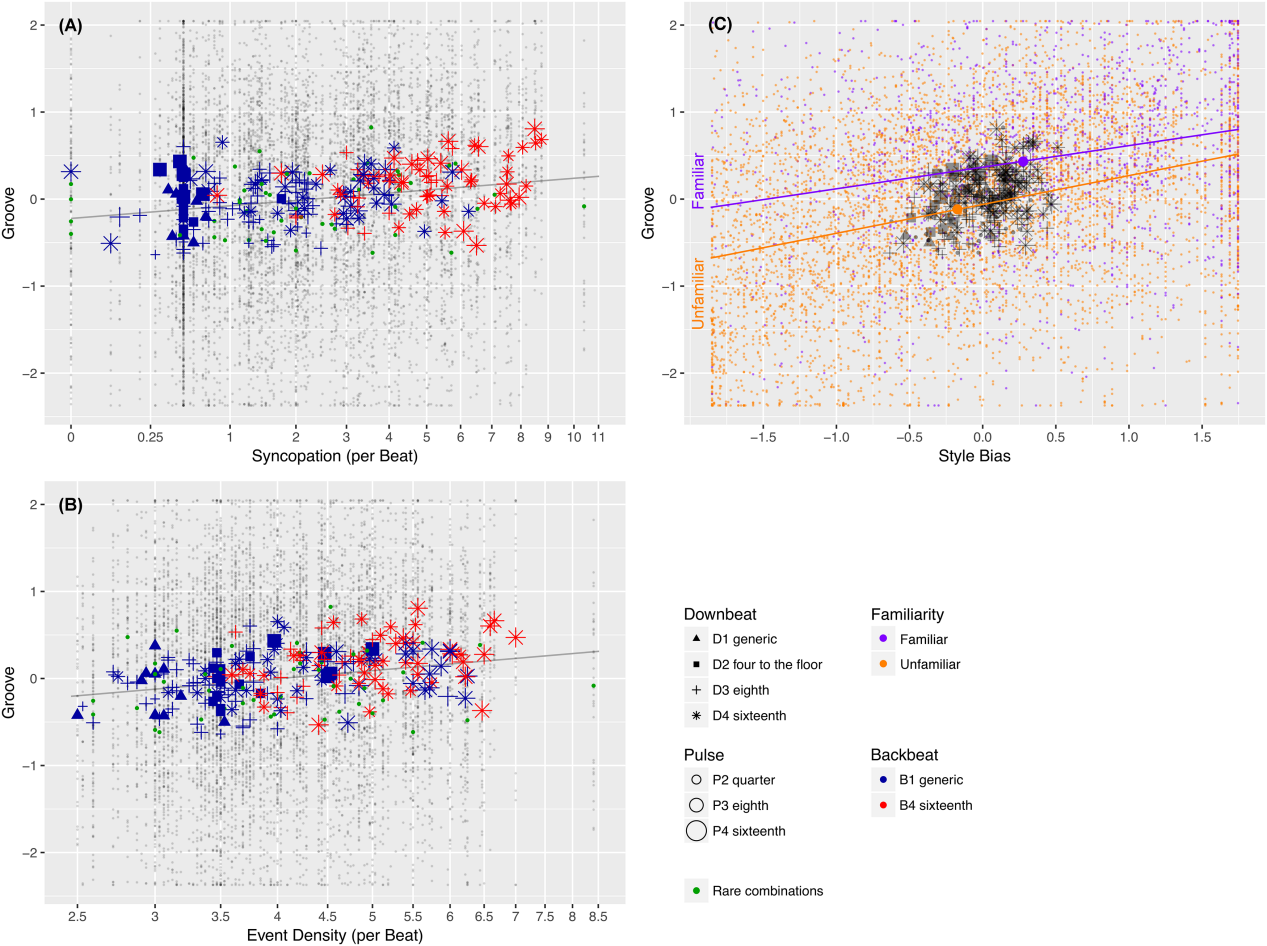
Syncopation, event density, style bias, familiarity. Groove ratings as a function of *Syncopation* (A), *Event Density* (B), *Style Bias* and *Familiarity* (C): Small dots denote single observations, large symbols denote the mean *Groove* ratings of the 248 stimuli. The symbol shapes, colours, and sizes represent the *Pattern Category* (see Fig 4). Sloping lines represent linear regression models.

There were weak associations between *Syncopation* and *Groove* (*R*^2^ = 0.010), and between *Event Density* and *Groove* (*R*^2^ = 0.011). The relationships can be inspected in Fig 5A and 5B. Note that the red, blue, and green symbols in the foreground represent mean *Groove* ratings for each stimulus, whereas the grey dots in the background represent single observations.

For both predictors, the associations with *Groove* were positive: *Groove* ratings tended to increase as *Syncopation* or *Event Density* increased. In either case, quadratic models did not have a significantly better fit with the data than simple linear models.

*Syncopation* and *Event Density* were mutually positively correlated (*r* = 0.500) with each other. Their combined effect (*R*^2^ = 0.013) on *Groove* is only slightly larger than the effect of each variable alone. We conclude that the two variables share much of their explanatory power for *Groove*. (Note that rows like *Event Density*×*Syncopation* in Table 4 refer to full multiple regression models, the effect sizes give the sum of all main effects and interactions).

Table 4 further shows that there was a significant interaction between *Syncopation* and *Expertise*. Combined, these predictors accounted for 1.6% of the *Groove* ratings’ variance (*R*^2^ = 0.016). Specifically, *Syncopation* had the strongest positive effect on *professional* musicians (*R*^2^ = 0.026, *p* < 0.001), a smaller positive effect on the *amateur* musicians (*R*^2^ = 0.009, *p* < 0.001), but no significant effect on the music *listeners* (*p* = 0.612).

Note that, on its own, *Expertise* explains only very little of the *Groove* ratings’ variance (*η*^2^ = 0.003, see Table 4). This means that, overall, the three *Expertise* groups’ *Groove* ratings did not differ much from each other, yet the three groups reacted differently to *Syncopation*.

A similar interaction can be observed for *Event Density* and *Expertise* (*R*^2^ = 0.016). The effect of *Event Denstity* was very small and positive for both the *listener* (*R*^2^ = 0.004, *p* = 0.005) and *amateur* (*R*^2^ = 0.006, *p* < 0.001) groups, but much larger and positive for the *professionals* (*R*^2^ = 0.029, *p* < 0.001). The combined effect of *Syncopation*, *Event Density*, and *Expertise* accounted for a total of 2.0% of the variance in the *Groove* variable (*R*^2^ = 0.020).

### Pattern category

**Fig 6:**
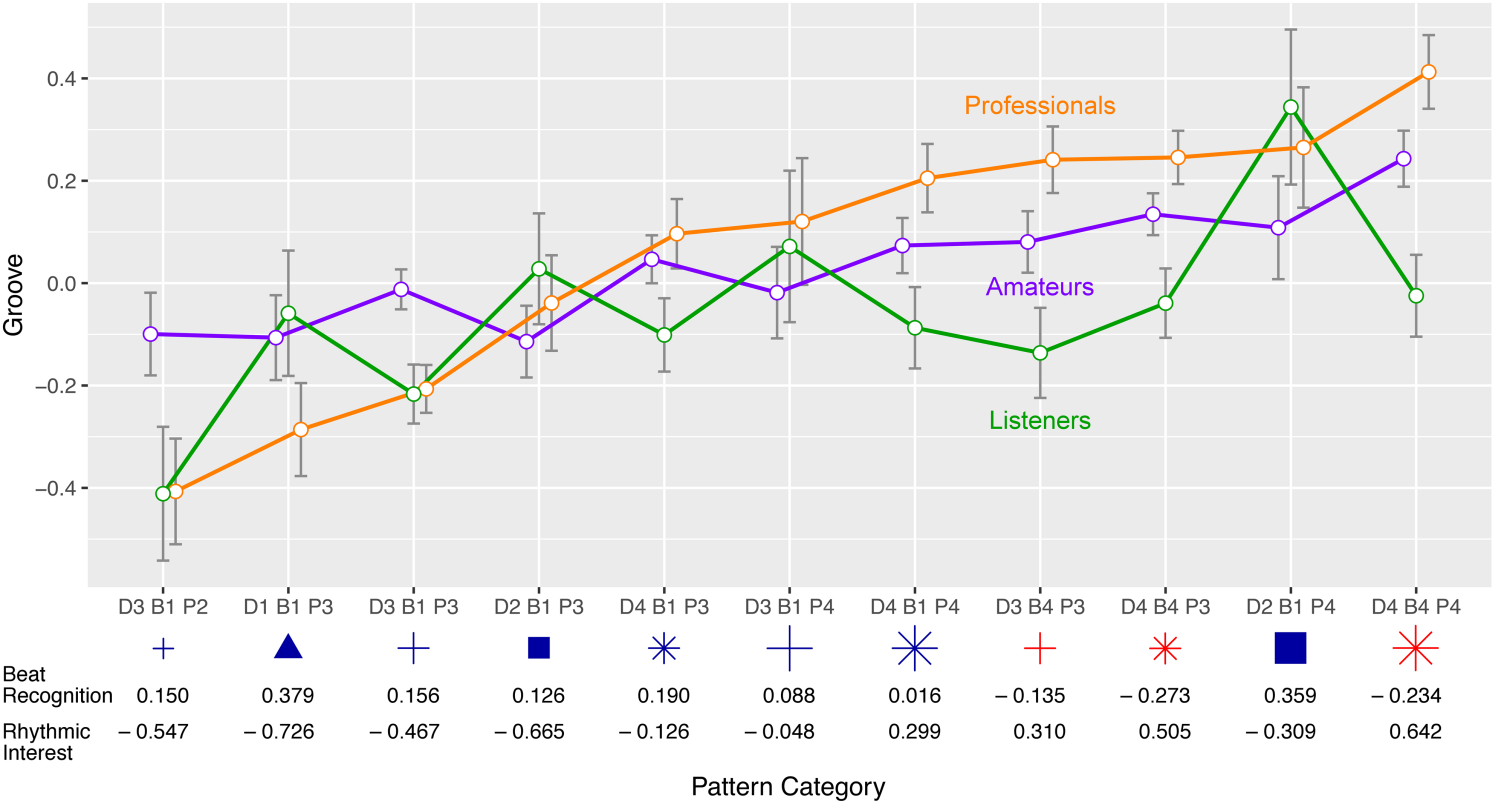
Pattern category × expertise interaction plot. *Groove* ratings as a function of *Pattern Category*, grouped by *Expertise*. Error bars are the standard error of the mean. Mean *Beat Recognition* and *Rhythmic Interest* ratings are given below each category.

The effect of *Pattern Category* was measured on the basis of a subset of the data that had been assigned to one of the eleven large *Pattern Categories* (200 stimuli with 6,673 observations). On its own, the *Pattern Category* variable explained 1.8% of the *Groove* variance (*η*^2^ = 0.018), slightly more than *Syncopation* and *Event Density* combined. In general, *Pattern Categories* with a 16^th^-note based *Backbeat* (*B4*, see red symbols in Figs 4 and 6) *Downbeat* (*D4*, stars) or *Pulse* layer (*P4*, large symbols) had a tendency to obtain a higher *Groove* response than their 8^th^-note- or quarter-note-based counterparts.

Mean *Beat Recognition* and *Rhythmic Interest* values are given in Fig 6 below each *Pattern Category*. We observe that mean *Groove* ratings are positively associated with mean *Rhythmic Interest*: categories with interesting patterns have also a tendency to be rated high on *Groove*. The association between mean *Beat Recognition* and *Groove* seems to be inverted: categories with easily understood patterns obtained lower mean *Groove* ratings than categories in which the beat was harder to track.

An interesting exception is the *D2 B1 P4* category (large blue squares): the small group of patterns with a four-to-the-floor *Downbeat*, a generic *Backbeat* and sixteenth notes in the *Pulse* (for an example, see No. 4 in Fig 3) are common in disco and electronic dance music. They obtained high *Groove* ratings, even though they were not considered to be rhythmically interesting by the participants.

We measured a significant and quite sizeable interaction effect between *Pattern Category* and *Expertise*: combined, the two variables explained 3.1% of the *Groove* ratings’ variance (*η*^2^ = 0.031). The interaction of the two predictors can be studied in Fig 6: *Pattern Category* had a substantially greater effect on the *professionals* (*η*^2^ = 0.054) than on the *amateurs* (*η*^2^ = 0.011) or *listeners* (*η*^2^ = 0.015).

The music *professionals* gave high *Groove* ratings to stimuli based on a sixteenth-notes grid in at least one of the rhythmic layers; their ratings peaked when all three layers of a pattern were based on sixteenth-notes (*D4 B4 P4*). These patterns were also those with a tendency to show high *Syncopation* and *Event Density* values (see Fig 5). *Professionals* associated rhythmically complex and dense patterns with high *Groove*, whereas simpler patterns obtained lower *Groove* ratings from the *professionals*.

*Amateur* musicians showed a flatter mean *Groove* response across the eleven categories than the *professionals*. The *amateurs*, like the *professionals*, had a tendency to give high *Groove* ratings to 16^th^-note based patterns, but they were less negatively influenced by patterns with larger subdivisions. Most notably, they did not give low ratings to the “archetypical rock pattern” *D1 B1 P3* (medium blue triangle) and to the only pattern category with a quarter-note based *Pulse*, *D3 B1 P2* (small blue cross), that is frequently used in rock and heavy metal.

The ratings of those participants who self-identified as mere music *listeners* were in many respects complementary to the *professionals*’ ratings. In general, *listeners* gave lower *Groove* ratings to the rhythmically more complex patterns with 16^th^-note based *Downbeat* and *Backbeat* layers, compared to the *professionals*. Conversely, *listeners* had a tendency to give relatively high ratings to categories with simple patterns, for example, the “archetypical rock pattern” *D1 B1 P3* (medium blue triangle), and the disco beats *D2 B1 P3* (medium blue squares) and *D2 B1 P4* (large blue squares).

### Familiarity and style bias

The binary *Familiarity* variable had a small-to-medium effect on the *Groove* ratings (*η*^2^ = 0.051, Table 4), exceeding all stimuli-related effects. Participants’s *Groove* ratings were higher when they thought they knew a pattern. The 1,780 *familiar* observations (i.e. observations for which participants signalled to know either the track or the band) obtained mean *Groove* ratings of 0.433, whereas the 6,549 *unfamiliar* observations had lower mean *Groove* ratings of −0.118. Participants considered the familiar patterns to also raise more *Rhythmic Interest* (0.162), compared to the unfamiliar patterns (−0.044). Participants further indicated that *Beat Recognition* was easier in familiar (0.268) compared to unfamiliar patterns (−0.066).

*Style Bias*, was positively associated with the *Groove* ratings (*r* = 0.351) and had a medium to large effect on *Groove* (*R*^2^ = 0.123). *Style Bias* was uncorrelated with *Beat Recognition* (*r* = 0.024), but positively associated with *Rhythmic Interest* (*r* = 0.262). So, participants gave high *Groove* ratings to stimuli they thought belong to a style they like, and they considered these patterns to be rhythmically interesting.

The combined effect of *Familiarity* and *Style Bias* qualifies as a large effect (*R*^2^ = 0.152) according to guidelines concerning the interpretation of effect sizes [113, 114]. This combined effect exceeds the effects of *Syncopation* or *Event Density* by a factor of approximately 15.

The effects of *Familiarity* and *Style Bias* can be studied in Fig 5C. This plot shows *Groove* as a function of *Style Bias*. Observations on stimuli that were *unfamiliar* to the participants are printed as small orange dots, whereas the observations on *familiar* stimuli are presented as small purple dots. (The mean values of the single stimuli on both *Style Bias* and *Groove* are presented grey in the background for context).

Linear regression models were fitted to the data, split according to the two *Familiarity* categories. The main effect of *Familiarity* shows in the offset between the two linear models: when participants thought “that they might know the band or the piece” (*familiar*) they had a tendency to give high *Groove* ratings (purple regression line). Conversely, *unfamiliar* stimuli obtained lower *Groove* ratings in the mean (orange regression line). *Style Bias* shows in the models’ slopes: participants rated stimuli high on *Groove* if they thought it belonged to a style they liked (high *Style Bias* values), and low if they disliked the style (low *Style Bias*).

*Familiarity* and *Style Bias* are not independent from each other: observations on *familiar* stimuli have a higher mean *Style Bias* (large purple dot in Fig 5C) than those on *unfamiliar* stimuli (large orange dot). This is no surprise: listeners are more likely to know a song or band from a style they appreciate (and presumably listen to more often) compared to a style they do not like.

Including *Expertise* as a third participant-related predictor did not significantly improve the model fit. The effect of *Style Bias* and *Familiarity* on *Groove* does not seem to depend on the *Expertise* of the participants.

## Discussion

This study measured the strength of participants’ *Groove* experience as a response to listening to reconstructions of Western popular music drum patterns. Fifteen stimuli- and participant-related variables were investigated as potential predictors for *Groove*. Most predictors were either related to participants’ perception of temporal regularities in the stimuli (which is thought to be a necessary condition for entrainment) or to the rhythmic interest triggered in the participants by the stimuli (which might in turn motivate bodily entrainment).

The results showed that the strongest predictors for *Groove* (*Syncopation*, *Event Density*, *Pattern Category*, *Familiarity*, *Style Bias*) were also positively associated with *Rhythmic Interest*. Listeners’ interest triggered by the music apparently was a relevant catalyst for groove.

Variables chosen due to their relation to *Beat Recognition* played a less important role in the study. This, however, does not mean that the perception of temporal regularities is irrelevant for groove: in this study, participants had a predominantly central European background, many of them were amateur or even professional musicians. Under these circumstances we can expect participants to easily grasp the temporal regularities of the music, and the precondition for entrainment (listeners understanding of these regularities) is generally satisfied.

Several variables had no or only tiny effects on *Groove*: *Residual Microtiming* (in accordance with [3, 62, 63], but conflicting with [64–69]), *Beat Salience* (in contrast to [3, 77]), *Pattern Variability*, and *Pattern Periodicity* were unrelated to *Groove*. We measured only very small effects for *Tempo*, *Tempo Change*, *Tempo Instability*, *8^th^ Note Swing*, and *16^th^ Note Swing*.

### Syncopation

We observed a generally positive relationship between *Syncopation* and *Groove*: more syncopated drum patterns had a tendency to obtain higher *Groove* ratings. This result resonates with the findings of Madison & Sioros [63] and Sioros et al. [73] who also concluded that *Syncopation* was positively associated with *Groove*. We have found evidence for an interaction between musical *Expertise* and *Syncopation*: for *professional* musicians, the positive correlation between *Syncopation* and *Groove* was greater than for *amateur* musicians; and the *listeners*’ ratings were not affected by *Syncopation*.

In their 2014 study, Witek et al. [5] hypothesised that the relationship between *Syncopation* and *Groove* might best be described by a ∩-shaped, curved model predicting a maximum *Groove* response for an intermediary level of *Syncopation*, and a lesser response for stimuli with either more or less *Syncopation*. Our data does not support this result: we found little evidence that a curved quadratic model with a maximum at a medium level of *Syncopation* had a better fit with the data than the simple first-order linear model.

We observed a much smaller overall effect size of *Syncopation* (*R*^2^ = 0.010) on *Groove* than Witek et al. They measured *R*^2^ = 0.347 for their *Move* response variable and *R*^2^ = 0.427 for *Pleasure* (see [5], p. 6). One important reason for this difference is that the statistical inference and the estimation of effect sizes in Witek et al. were based on the mean ratings of the 50 stimuli. The within-stimuli variance caused by participants’ disagreement about the stimuli was discarded before the analysis. In the present study, statistical inference is based on all observations and a large portion of the variance comes from disagreement among the participants about the groove qualities of the same stimuli. The *Stimuli* only explained 9.6% of the *Groove* ratings’ total variance (see Table 4), leaving more than 90% to disagreement among participants.

In order to compare our results with those reported by Witek et al. [5], we carried out a post-hoc linear regression analysis on the means of the stimuli, with *Syncopation* as explanatory variable. *Syncopation* accounted for 15% of the variance among the 248 stimuli’s mean *Groove* ratings (*R*^2^ = 0.145). This amounts to a bit less than half the effect that Witek et al. [5] reported for the *Move* variable, and about a third of the effect they measured for *Pleasure*.

### Event density

*Event Density* was positively associated with *Groove*, which confirms previous findings by Madison et al. [3]. Potentially, however, the effect measured in our study might be exaggerated: the stimuli present the drum patterns in isolation by omitting the rest of the band’s music. In consequence, some patterns may sound empty or incomplete because important complementary instrumental layers are missing (like a bass line or a guitar riff). It is likely that the patterns with higher *Event Density* obtained higher *Groove* ratings, because they sounded fuller and more complete.

The effect of *Event Density* (similar to *Syncopation*) was substantially moderated by musical *Expertise*: music *professionals*’ *Groove* ratings reacted more positively to stimuli with high *Event Density*, compared to the ratings of the *amateur* and *listener* groups. As in the case of *Syncopation*, *Event Density* does not seem to be a universal musical factor that increases the *Groove* experience for all (or at least most) listener groups. Rather the effect of *Event Density* depends on *Expertise*, a participant-related factor.

### Pattern category

The *Pattern Category* variable accounts for certain aspects of a drum pattern’s musical composition or arrangement. These features are by far more basic than the properties described in the musicological and ethnomusicological literature on the structure of “grooves” as multi-layered rhythmic patterns [9, 10, 12, 14]. Yet, in spite of its simplicity, the *Pattern Category* predictor explained a non-negligible portion of the variance in the *Groove* ratings. This indicates that it might be worthwhile studying aspects of composition and arrangement more thoroughly in order to better understand musical factors influencing the groove experience.

Again, musical *Expertise* plays a relevant moderating role: music *professionals* had a tendency to give high *Groove* ratings to *Pattern Categories* that allow for greater rhythmic complexity (Table 4, Fig 6). *Amateur* musicians and *listeners* on the other hand were more ready to give high ratings to categories with simpler rhythmic patterns.

The moderating effect of *Expertise* on *Groove* is a recurring theme in this study: all three major music-related variables of this study (*Syncopation*, *Event Density*, *Pattern Category*) seem to depend on *Expertise*.

### Familiarity and style bias

We measured relatively large effects of *Familiarity* and *Style Bias* on the experience of *Groove*: people gave high groove ratings to music they thought they knew and to music they thought belongs to a style they liked.

The notion that listeners’ musical taste affects their *Groove* response agrees with common sense. It is reasonable to assume that listeners’ susceptibility to bodily entrainment as a response to music is strengthened when the music agrees with their taste. Since listeners’ activities and preferences differ substantially, it is no surprise that the same stimulus might trigger diverse groove reactions in different people. Consequently, we would expect listeners’ *Style Bias* to play a role in moderating their groove response.

Also, we may expect that listeners’ *Familiarity* with a stimulus is relevant to its *Groove* effect. In a recent study, Madison & Schiölde [82] showed that repeated exposure increases listeners’ liking of a musical stimulus. This, to a certain degree, is equivalent to increasing the pleasure someone experiences while listening. Assuming that repeated listening is an activity that increases listeners’ *Familiarity* with a stimulus, our data suggests that a similar claim holds for *Groove*.

Yet, it came as a surprise that these two listener-related factors were so much more important for *Groove* than the most relevant music-related factors in the study. The combined participant-related effects of *Style Bias* and *Familiarity* (*R*^2^ = 0.152) exceeded the largest purely stimuli-related factor (*Pattern Category*, *η*^2^ = 0.018) by almost a magnitude.

This was even more surprising considering that the stimuli consisted of drum pattern reconstructions only. The stimuli lacked much of the musical information (melody, instrumentation, lyrics, timbre) that helps participants recognise the music and its style. We must assume that participants had only weak style associations while listening to the reconstructed drum patterns. These associations are likely to be stronger when participants are confronted with the original music, which offers more explicit clues to associate a stimulus with a song or a style. Hence, the effects of *Style Bias* and *Familiarity* on *Groove* might be even stronger when participants are confronted with the original recordings.

The massive size difference between music- and listener-related effects raises the question whether some aspect of the experimental design led to an overstatement of the listener-related effects. For example, participants were asked about style and familiarity every time they listened to a stimulus. The simple fact that participants thought about style and familiarity repeatedly and in close proximity to the ratings might have emphasised the associations between the *Groove* and the *Style Bias* and *Familiarity* variables. (Such an effect might be similar to the *Proximity Effect* [100] or the *Recency Effect* observed in the study of memory [115, 116]).

Two further listener-related factors (beyond *Familiarity*, *Style Preference*, and *Expertise*) have not been considered in this study, yet they probably have a considerable impact on groove: listeners’ positive attitude to dancing (or other activities that require bodily entrainment) is likely to coincide with a strong groove response, as shown by Witek et al. [5]. And listeners’ open-earedness [117] might be relevant as well: open and curious listeners will probably be more responsive to previously unknown music than narrow-minded listeners.

## Conclusions

This study aimed at investigating how fifteen music- and participant-related predictors covary with listeners’ groove experience. In the past, groove research predominantly focused on music-related factors. Our findings suggest that listener-related predictors like taste and familiarity play a relevant role in shaping the groove experience. They also suggest that musical expertise strongly moderates the effect of music-related factors on groove.

If these findings prove to be consistent in future research, they challenge the validity of what we believed to be a core idea of groove studies: namely the notion that there are musical factors which universally affect groove in a general population of listeners. Our results raise the question to what extent such universal musical factors exist. We hypothesise that specific musical factors trigger groove to a different extent across groups of people that are characterised by their members’ attitudes towards music.

Groove research, including our own, has investigated a multitude of potential factors that influence groove. Many results (especially on *Syncopation* or *Microtiming*) seem to contradict each other. The studies used different methodologies and investigated different repertoires, which might account for some of the discrepancies. But the studies also sampled their participants from different populations with specific cultural backgrounds. The differences among the surveyed populations might additionally account for a relevant portion of the discrepancies.

This suggests to further expand the focus of groove research in the future. In addition to studying primarily musical factors and their impact on listeners, we may recognise the listeners themselves and their interaction with music as relevant topics of our investigation. Conclusions on the effects of musical properties on groove will rest on more solid foundations if we know which music the participants usually listen to, which music they appreciate, which music they have grown up with, which music accompanies their dancing and workouts, how well they are informed about music, which music they practise, how competently they practise it, and how open they are to engage with music they have never heard before. This implies collecting comprehensive information about the individuals who participate in our studies.

For some studies it might be sensible to select a homogenous sample of participants who share style preferences and biographic details: we expect to see that musical factors which influence groove in a certain stylistic context are most effective within a group of listeners who are attracted to and familiar with this style. We plan to investigate this topic in a future paper that will be based on the present study’s data: we will divide the participants into groups with more or less homogenous style preference profiles (using exploratory factor analysis), and discuss the effect of style-specific musical properties within these groups.

Conversely, if a study investigates whether a factor has an universal effect in a more general population, it might be necessary to stratify the sample of participants according to proportions in the target population for better representativeness.

## Supporting information

S1 Table List of drummers and tracks. (XLSX)
